# Systematic review of Kaixinsan in treating depression: Efficacy and pharmacological mechanisms

**DOI:** 10.3389/fnbeh.2022.1061877

**Published:** 2022-12-06

**Authors:** Menghan Bo, Hongjing Zhang, Jia Xu, Hong Zhao, Xinglei Jia, Guangdong Wang, Zhengyu Lu

**Affiliations:** ^1^VIP Department, Longhua Hospital, Shanghai University of Traditional Chinese Medicine, Shanghai, China; ^2^Teaching Affairs Department, Yueyang Hospital of Integrated Traditional Chinese and Western Medicine, Shanghai University of Traditional Chinese Medicine, Shanghai, China; ^3^Institute of Chinese Materia Medica, Shanghai University of Traditional Chinese Medicine, Shanghai, China

**Keywords:** Kaixinsan, depression, traditional Chinese medicine prescription, network pharmacology, efficacy

## Abstract

**Introduction:**

Kaixinsan (KXS) has been in use as an effective classic formulation of traditional Chinese medicine for depression. However, its active components and action mechanism against depression remain elusive. The purpose of this study was to summarize and evaluate the efficacy and potential pharmacological mechanisms of KXS in antidepressant treatment.

**Materials and methods:**

Reports on the use of KXS in the treatment of depression were systematically collected from PubMed, Web of Science, Embase, China National Knowledge Infrastructure, Chongqing VIP, and Wanfang Data from the establishment to July 2022, including those on mood disorders in neurological diseases such as Alzheimer’s disease. Meta-analysis was conducted with the Review Manager 5.3 software. Online datasets, traditional Chinese medicine system pharmacological analysis platform, GeneCards, online Mendelian inheritance in man, and DisGeNET were used to investigate the depression-related genes. The gene ontology (GO) and Kyoto Encyclopedia of Genes and Genomes (KEGG) pathway enrichments were performed to construct the ‘component-target-pathways’ network using Metascape online analyses.

**Result:**

Ten studies were included in the analysis. Meta-analysis showed that both low-dose KXS (SMD = 19.66, *Z* = 7.96, and *I*^2^ = 42%) and high-dose KXS (SMD = 23.84, *Z* = 8.46, and *I*^2^ = 13%) could increase the sucrose preference in depression models. In addition, 5-hydroxytryptamine (5-HT) (SMD = 10.91, *Z* = 2.95, and *I*^2^ = 50%) returned to normal level after the treatment at low dose KXS. In network pharmacology, 50 active components and 376 gene targets were screened out. AKT1, GAPDH, ALB, TNF, and TP53 were the core target proteins. GO analysis showed that KXS mainly treats depression in biological processes such as response to drugs, cellular calcium ion homeostasis, and regulation of chemical synaptic signal transmission. KEGG results show that the mechanism of action of KXS in treating depression is through neural activity ligand-receptor interaction, the calcium signaling and CAMP signaling pathways.

**Discussion:**

The study reveals the active components and potential molecular mechanism of KXS in the treatment of depression and provides evidence for future basic research.

## Introduction

In recent years, numerous studies have shown the need for increased attention to mental health (Pan et al., 2021). Deaths caused by mental disorders account for 14.3% of global mortality (Walker et al., 2015), and depression is one of the most common mental disorders. Depression is characterized by loss of pleasure, fatigue, and worthlessness, as well as cognitive impairment and neurotrophic symptoms including anorexia, memory alterations, and sleep disturbances (Ribeiro et al., 2018). To date, little is known about the etiology of depression. A sequence of stressful events leads to negative physical and psychological effects, and such changes are tightly linked with the depression (Yang et al., 2015). Depression is often insidious and unpredictable (Malhi and Mann, 2018). One reason is that some depressed patients choose to conceal their mental symptoms to avoid being stigmatized (Dubovsky et al., 2021). Patients with other frequently occurring diseases are at high risk of depression (Read et al., 2017); however, there is the possibility of focusing on the underlying disease and ignoring depressive symptoms. The elderly are at risk of frequently occurring diseases due to reduced physical activity and worsening health status (Reynolds et al., 2019). In addition, bereavement, living alone, lack of social attention, lack of sleep (Casey, 2017), and aging have been identified as independent risk factors for depression. Depression in older adults often accompanies cognitive impairment, making it more difficult to identify (Linnemann and Lang, 2020). Depression increases the risk of obesity, coronary artery disease, and inflammation (Aksay et al., 2016; Biffi et al., 2017; Leonard, 2018). At present, western medicine antidepressants mainly include the following categories: monoamine oxidase inhibitors (MAOIs), tricyclic antidepressants (TCAs), and serotonin reuptake inhibitors (SSRIs). However, chemical drugs are often accompanied by clinical problems such as adverse reactions and drug resistance (Willner et al., 2014). There were nearly half of the patients still failed to respond after receiving multiple antidepressant treatments, or relapse again due to trivial matters, which is not conducive to recovery of the patients and increases the possibility of developing refractory depression (Belzung, 2014; Dodd et al., 2021).

Traditional Chinese medicine prescriptions composed of multiple herbs can act on different targets at the same time and have long been found to be effective in health care and disease treatment (Zhang et al., 2013). Kaixinsan (KXS), recorded in Sun Simiao’s “Prescriptions for Emergencies” in the Tang Dynasty, was used to treat dementia and amnesia; other herbs include Ginseng Radix Rhizoma (GR; root and rhizome of Panax ginseng C. A. Mey.), Polygalae Radix (PR; root of Polygala tenuifolia Wild.), Acori Tatarinowii Rhizoma (ATR; rhizome of Acorus tatarinowii Schott), and Poria [PO; sclerotium of Poriacocos (Schw.) Wolf]. The ratio of the four herbs in the prescription is 1:1:25:50. Modern clinical studies have shown that KXS has significant efficacy in the treatment of depression associated with multiple causes (Fu et al., 2019; Hu et al., 2021a,b).

Although KXS has been proven to be a classical Chinese medicine compound with a positive effect on depression, the underlying mechanism of its effect has not been fully elucidated. A meta-analysis, combined with network pharmacology, was used in this study to systematically evaluate the efficacy and pharmacological mechanism of KXS for depression. The results will promote further research on the treatment of depression.

## Materials and methods

### Meta-analysis to evaluate the efficacy of Kaixinsan in the treatment of depression

The reports on experiments on the use of KXS in the treatment of depression were systematically collected from PubMed, Web of Science, Embase, China National Knowledge Infrastructure (CNKI), Chongqing VIP (CQVIP), and Wanfang Data from the establishment to July 2022, the searched keywords were kaixinsan, kai-xin-san, depression, mood disorders. As depression is one of the main psychiatric symptoms of AD, AD models with depressive symptoms were also included in the analysis. See Supplementary material for the search strategy.

#### Inclusion criteria

Studies on animal models of depression were included in the analysis, and there were no restrictions on animal species, sex, age, and weight. KXS is only used as an intervention drug, and there is no limit to its dosage. Other positive therapeutic drugs and Chinese herbal medicine prescriptions were not added; the control group was treated with non-therapeutic liquid (normal saline or distilled water); the evaluation of the outcome after treatment was based on the index level of depressive function or mechanism.

#### Exclusion criteria

Reviews, conference abstracts, non-animal studies, studies incorporating other traditional Chinese medicine prescriptions, and duplicate articles were excluded.

#### Article screening and data extraction

All articles were screened and included by two independent researchers. Relevant information was extracted from the included studies, including authors, year of publication, sex, age, weight, number, dose of KXS, and data on outcome assessment of experimental animals. Getdate software was used to extract the data for graphical representation.

#### Statistical analysis

Review Manager 5.3 was used for the meta-analysis. Two independent researchers used the SYRCLE Animal experimental bias risk assessment tool to evaluate the included articles, and any disagreements were resolved through consultation. The SYRCLE animal experiment bias risk assessment tool was for animal experiment assessment, which was established on the basis of the Cochrane bias risk assessment tool. It included 10 items in total, among which baseline characteristics, randomization of animal placement, and assessment of random results were new items. In the results, data such as the sucrose preference index in the sucrose preference test, and 5-HT levels were treated as continuous data. Standard mean difference (SMD) and 95% confidence interval (CI) were determined using Review Manager 5.3. A random effects model was also used on the data to estimate combined effect sizes, the Q statistic was used to assess heterogeneity, and the *I*^2^ statistic was used to quantify the magnitude of heterogeneity.

### Research on Kaixinsan in the treatment of depression based on network pharmacology

#### Network pharmacology data preparation

##### Kaixinsan drug active ingredient screening and target acquisition

The traditional Chinese medicine system pharmacological analysis platform (TCMSP)^1^ was used to collect the traditional Chinese medicine ingredients of GR, PO, and ATR, and the oral bioavailability (OB) was set to be ≥ 30%. The drug-likeness (DL) was ≥ 0.18 (Pang et al., 2018) and the active ingredients of traditional Chinese medicine were obtained. The active ingredients of PR were obtai ned at the herb station.

The compounds of KXS obtained from the TCMSP database were used to obtain the action targets of GR, PO, and ATR according to MOL ID. The effective components of PR were imported into the Pubchem database,^2^ and the components SMILES (simplified molecular input line entry system) and the chemical structural formula were obtained. In the same line, the structural formula was imported into the database, swisstargetprediction^3^ to predict the target. The UniProt^4^ database was used to download the compound data table. The “TRIM” function is used to optimize the data, the “VLOOKUP” function was used to match the target gene name. We supplemented the unmatched gene names by consulting the literature. The relevant target proteins of the chemical components obtained by the above methods were annotated using UniProt.

##### Prediction of disease targets

GeneCards,^5^ online Mendelian inheritance in man (OMIM),^6^ and Disgenet^7^ platforms were used to obtain disease-related targets. “Depression” was used as the keyword to search for depression-related targets. Setting the disease object as “human,” the “VLOOKUP” function was used to match the target gene names and screen out the intersection genes of drugs and diseases.

##### Key target acquisition

The Venny^8^ software was used to obtain the intersection targets of the active compounds of herbs and depression as potential targets for the treatment of depression in traditional Chinese medicine.

#### Network analysis and protein-protein interaction construction

##### Traditional Chinese medicine network visualization

The four traditional Chinese herbs, their drug-related active chemical components, and the predicted target gene were imported into Cytoscape (V3. 7. 2) software. Using the degree of analysis of the imported data, the protein interaction network diagram of traditional Chinese medicine, active ingredients, and predicted targets were formed.

##### Constructing a protein interaction analysis network diagram

The intersection genes were input to the String^9^ platform, setting the object as “homo sapiens,” the highest confidence score was 0.9, and the free gene nodes were hidden to obtain the protein-protein interaction (PPI) relationship. The results were imported into Cytoscape 3.7.2, and “network analyze” was selected to obtain the network topology parameters.

Degree referred to the interconnection between proteins. Betweenness centrality (BC) reflects the number of times the shortest path passes through a single node. Closeness centrality (CC) means the ease of communication between nodes.

#### Gene ontology and Kyoto encyclopedia of genes and genomes enrichment analysis

Gene ontology (GO) enrichment and KEGG (Kyoto encyclopedia of genes and genomes) helped explain the biological process of the target and its role in metabolism and signal transduction. Drug-disease targets were input into the String database and DAVID database^10^ to obtain the GO enrichment and KEGG pathway. The species was restricted to Homo sapiens, and only the items with a *p*-value < 0.05 were displayed. The bubble diagram was drawn using R software.

## Results

### Meta-analysis

**FIGURE 1:**
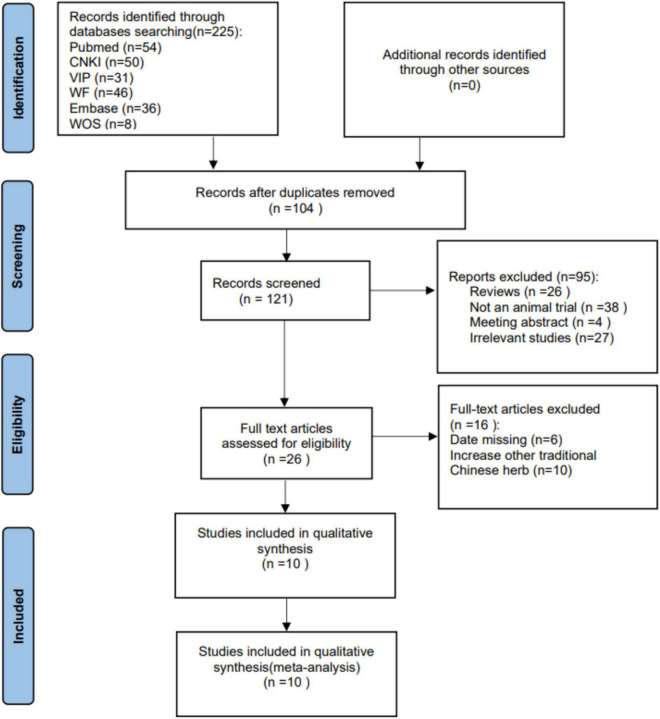
The PRIMSA flow diagram of study selection.

**FIGURE 2:**
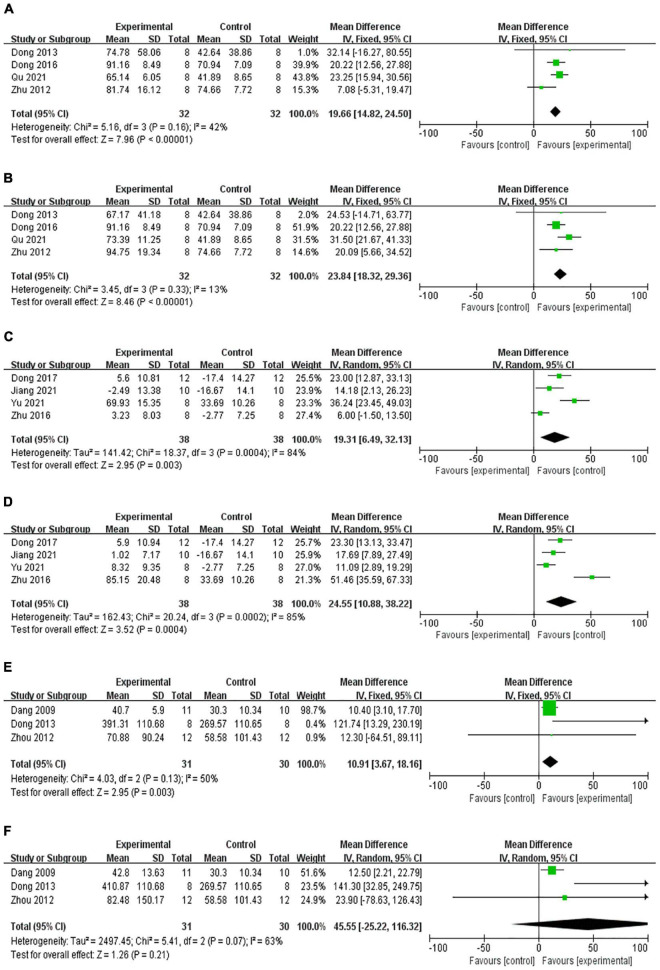
Forest plot shows that low-dose **(A)** and high-dose **(B)** KXS increased the sucrose consumption in the depression model, compared with sucrose consumption before and after treatment with KXS, which shows a statistically significant increase in sucrose preference at both low-dose **(C)** and high-dose **(D)**. Low-dose KXS could increase the 5-HT levels **(E)**, not statistically significant in the 5-HT level following high-dose KXS **(F)**.

After screening, 10 out of 225 studies (Table 1) were included in the analysis (Dang et al., 2009; Zhou et al., 2012; Zhu et al., 2012, 2016; Dong et al., 2013, 2016, 2017; Jiang et al., 2021; Qu et al., 2021; Yu et al., 2021; Figure 1). As one of the indexes of depression function, eight studies measured sucrose preference in depression model rats. Four studies analyzed the outcome of sucrose consumption after treatment with KXS (Zhu et al., 2012; Dong et al., 2013, 2016; Qu et al., 2021). The results show that both low-dose KXS (*P* < 0.00001, SMD = 19.66, 95% confidence interval [14.82, 24.50], heterogeneity: *x*^2^ = 5.16, *I*^2^ = 42%, Figure 2A) and high-dose KXS (*P* < 0.00001, SMD = 23.84, 95% confidence interval [18.32, 29.36], heterogeneity: *x*^2^ = 3.45, *I*^2^ = 13%, Figure 2B) could increase sucrose consumption of depression models. In the same line, four studies evaluated sucrose preference in depression models before and after KXS treatments (Zhu et al., 2016; Dong et al., 2017; Jiang et al., 2021; Yu et al., 2021), both low-dose KXS (*P* = 0.003, SMD = 19.31, 95% confidence interval [6.49,23.13], heterogeneity: *x*^2^ = 18.37, *I*^2^ = 84%, Figure 2C) and high-dose KXS (*P* = 0.0004, SMD = 24.55, 95% confidence interval [10.88, 38.2], heterogeneity: *x*^2^ = 20.24, *I*^2^ = 85%, Figure 2D) showed positive effects. Four studies reported levels of 5-hydroxytryptamine (5-HT) (Dang et al., 2009; Zhou et al., 2012; Zhu et al., 2012; Dong et al., 2013). The data showed that after using low-dose KXS (*P* = 0.003, SMD = 10.91, 95% confidence interval [3.67, 18.16], heterogeneity: *x*^2^ = 4.03, *I*^2^ = 50%, Figure 2E) 5-HT levels recovered to normal level, but when high-dose KXS was used to assess the level of 5-HT in the depression models, there was no significant difference between the treatment group and the control group (*P* = 0.21, SMD = 45.55, 95% confidence interval [−25.22, 116.32], heterogeneity: *x*^2^ = 5.41, *I*^2^ = 63%, Figure 2F). The low risk rate was 42%, the medium risk rate was 52%, and the high risk rate was 6% in all items of animal experiment evaluation. All studies provided brief study details, the number and total of animals in each group were listed, and randomization was used for grouping, but no further method was specified. In terms of animal experiments, nine studies have described the animal species, only one study has provided age, but all studies have provided the animal weight and gender. During the experiment, each study recorded the experimental method and time, but lacked a specific description of the principle (Table 2).

**TABLE 1:** Characteristics of the 10 included studies.

References	Species (*n* = experimental/ control)	Sex (age)	Weight	Intervention for the experimental group	Central group	Outcome measure
Zhu et al. (2012)	SD rats (*n* = 8/8)	Male (NA)	200–220 g	KXS, i.g.0.9 g/2.7 g/kg/day	Normal saline	Sucrose preference index in sucrose preference test; 5-HT
Qu et al. (2021)	/(*n* = 8/8)	Male (NA)	22–25 g	KXS, i.g.3/10 g/kg/day for 7 days	Normal saline	Sucrose preference index in sucrose preference test
Dong et al. (2013)	Wistar rats (*n* = 8/8)	Male (NA)	180 ± 10 g	KXS, i.g.338/676 mg/kg/day for 4 weeks	Distilled water	Sucrose preference index in sucrose preference test; 5-HT
Dong et al. (2016)	Wistar rats (*n* = 8/8)	Male (6 weeks)	180 ± 10 g	KXS, i.g.370 mg/kg/day for 7 weeks	Distilled water	Sucrose preference index in sucrose preference test
Jiang et al. (2021)	SD rats (*n* = 10/10)	Male (NA)	180–200 g	KXS, i.g.67.5/270 mg/kg/day for 7 days	Normal saline	Sucrose preference index in sucrose preference test
Yu et al. (2021)	Wistar rats (*n* = 8/8)	Male (NA)	180–220 g	KXS, i.g.338/676 mg/kg/day for 3 weeks	Distilled water	Sucrose preference index in sucrose preference test
Dong et al. (2017)	Wistar rats (*n* = 12/12)	Male (NA)	200 ± 10 g	KXS, i.g.1.5/5 g/kg/day for 7 days	Normal saline	Sucrose preference index in sucrose preference test
Zhu et al. (2016)	Wistar rats (*n* = 8/8)	Male (NA)	200–220 g	KXS, i.g.0.3/2.7 g/kg/day for 5 weeks	Normal saline	Sucrose preference index in sucrose preference test
Dang et al. (2009)	SD rats (*n* = 10/10)	Male or female (NA)	190–250 g	KXS, i.g.175/350 mg/kg/day for 7 days	Distilled water	5-HT
Zhou et al. (2012)	Kunming mice (*n* = 12/12)	Male (NA)	21–30 g	KXS, i.g.370 mg/kg/day for 7 weeks	Normal saline	5-HT

**TABLE 2:** Evaluation results of SYRCLE animal experiment risk assessment tool.

Type of bias	Selection bias			Performance bias		Detection bias		Attrition bias	Reporting bias	Other	Total
References	Sequence generation	Baseline characteristics	Allocation concealment	Random housing	Blinding	Random outcome assessment	Blinding	Incomplete outcome data	Selective outcome reporting	Other sources of bias	
Zhu et al. (2012)	1	1	1	2	1	2	1	0	1	2	12
Qu et al. (2021)	1	1	1	2	1	1	2	0	2	2	13
Dong et al. (2013)	1	1	1	2	1	2	2	0	1	2	13
Dong et al. (2016)	1	1	2	2	1	2	2	0	1	2	14
Jiang et al. (2021)	1	2	1	1	1	2	2	1	2	2	15
Yu et al. (2021)	1	2	1	1	2	1	2	1	2	2	15
Dong et al. (2017)	1	2	1	2	1	2	2	1	2	1	14
Zhu et al. (2016)	1	2	1	2	2	1	1	2	2	1	15
Dang et al. (2009)	1	1	1	1	1	2	2	0	2	2	13
Zhou et al. (2012)	1	1	1	2	1	1	1	0	1	2	11
The rate of “low risk”	0%	40%	10%	70%	20%	60%	70%	10%	60%	80%	–

2, Low risk; 1, Uncertain risk; 0, High risk.

### Network pharmacology

Pharmacokinetics is the process of studying the absorption, distribution, metabolism, and excretion (ADME) of drugs in the body. Based on the ADME index (OB ≥ 30%, DL ≥ 0.18), 60 compounds of KXS were selected, including 22 species of GR, 15 species of PO, 4 species of ATR (Table 3), and 19 species of PR (Table 4). After removing non-targeted compounds, 46 active compounds were finally included in the analysis.

**TABLE 3:** Compounds of Ginseng Radix Rhizoma (GR), Poria (PO), and Acori Tatarinowii Rhizoma (ATR).

MOL ID	Molecule name	OB (%)	DL (%)	Conversion ID
MOL005348	Ginsenoside-Rh4_qt	31.11	0.25	RS1
MOL005357	Gomisin B	31.99	0.19	
MOL005376	Panaxadiol	33.09	0.22	RS2
MOL005344	Ginsenoside rh2	36.32	0.24	RS3
MOL000358	Beta-sitosterol	36.91	0.23	RS4
MOL005399	alexandrin_qt	36.91	0.23	RS5
MOL004492	Chrysanthemaxanthin	38.72	0.3	
MOL005317	Deoxyharringtonine	39.27	0.23	RS6
MOL005401	Ginsenoside Rg5_qt	39.56	0.24	
MOL005318	Dianthramine	40.45	0.42	RS7
MOL000422	Kaempferol	41.88	0.24	RS8
MOL002879	Diop	43.59	0.28	RS9
MOL000449	Stigmasterol	43.83	0.22	RS10
MOL005320	Arachidonate	45.57	0.26	RS11
MOL005384	Suchilactone	57.52	0.28	RS12
MOL005360	Malkangunin	57.71	0.3	
MOL000787	Fumarine	59.26	0.3	RS13
MOL005356	Girinimbin	61.22	0.33	RS14
MOL003648	Inermin	65.83	0.3	RS15
MOL005321	Frutinone A	65.9	0.47	RS16
MOL005308	Aposiopolamine	66.65	0.35	RS17
MOL005314	Celabenzine	101.88	0.35	
MOL000273	(2R)-2-[(3S,5R,10S,13R,14R,16R,17R)-3,16-dihydroxy-4,4,10,13,14-pentamethyl-2,3,5,6,12,15,16,17-octahydro-1H-cyclopenta[a]phenanthren-17-yl]-6-methylhept-5-enoic acid	30.93	0.81	FL1
MOL000275	Trametenolic acid	38.71	0.8	FL2
MOL000276	7,9(11)-dehydropachymic acid	35.11	0.81	
MOL000279	Cerevisterol	37.96	0.77	FL3
MOL000280	(2R)-2-[(3S,5R,10S,13R,14R,16R,17R)-3,16-dihydroxy-4,4,10,13,14-pentamethyl-2,3,5,6,12,15,16,17-octahydro-1H-cyclopenta[a]phenanthren-17-yl]-5-isopropyl-hex-5-enoic acid	31.07	0.82	
MOL000282	Ergosta-7,22E-dien-3beta-ol	43.51	0.72	FL4
MOL000283	Ergosterol peroxide	40.36	0.81	FL5
MOL000285	(2R)-2-[(5R,10S,13R,14R,16R,17R)-16-hydroxy-3-keto-4,4,10,13,14-pentamethyl-1,2,5,6,12,15,16,17-octahydrocyclopenta[a]phenanthren-17-yl]-5-isopropyl-hex-5-enoic acid	38.26	0.82	
MOL000287	3beta-Hydroxy-24-methylene-8-lanostene-21-oic acid	38.7	0.81	
MOL000289	Pachymic acid	33.63	0.81	
MOL000290	Poricoic acid A	30.61	0.76	
MOL000291	Poricoic acid B	30.52	0.75	
MOL000292	Poricoic acid C	38.15	0.75	
MOL000296	Hederagenin	36.91	0.75	FL6
MOL000300	Dehydroeburicoic acid	44.17	0.83	
MOL003542	8-Isopentenyl-kaempferol	38.04	0.39	SCP1
MOL003576	(1R,3aS,4R,6aS)-1,4-bis(3,4-dimethoxyphenyl)-1,3,3a,4,6,6a-hexahydrofuro[4,3-c]furan	52.35	0.62	SCP2
MOL003578	Cycloartenol	38.69	0.78	SCP3
MOL000422	Kaempferol	41.88	0.24	SCP4

**TABLE 4:** Compounds of Polygalae Radix (PR).

Ingredient ID	Ingredient name	Conversion ID
HBIN041726	Quercitrin	YZ1
HBIN040415	Polygalaxanthone iii	YZ2
HBIN040089	Piperolactam a	YZ3
HBIN020976	Citral	YZ4
HBIN042149	Reynoutrin	YZ5
HBIN033265	Linalool	YZ6
HBIN031102	Isoquercitrin	YZ7
HBIN045073	Sucrose	YZ8
HBIN015652	Alpha-pinene	YZ9
HBIN046387	Thymol	YZ10
HBIN011118	5,6,7-Trimethoxycoumarin	YZ11
HBIN007432	3,4-Dimethoxy cinnamic acid	YZ12
HBIN037343	Norharman	YZ13
HBIN029831	Hyperin	YZ14
HBIN002968	1-Peroxyferolide	YZ15
HBIN028801	Harman	YZ16
HBIN001869	1,6-Dihydroxy-3,7-dimethoxyxanthone	YZ17
HBIN032827	Leaf alcohol	YZ18
HBIN033245	Limonene	YZ19

**FIGURE 3:**
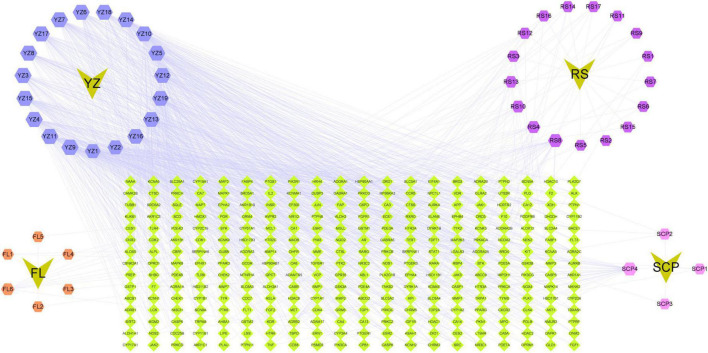
The inverted triangle represented the constituent drugs of the compound KXS, the regular hexagon represented the active ingredient of each drug, and the diamond represented the predicted target of each active ingredient. RS, Ginseng Radix Rhizoma (GR; root and rhizome of Panax ginseng C. A. Mey.); FL, Poria [PO; sclerotium of Poriacocos (Schw.) Wolf]; YZ, Polygalae Radix (PR; root of Polygala tenuifolia Wild.); SCP, Acori Tatarinowii Rhizoma (ATR; rhizome of Acorus tatarinowii Schott).

The relationship between each herb and the predicted target in KXS (Figure 3). The inverted triangle represented the constituent drugs of the compound KXS, the regular hexagon represented the active ingredient of each drug, and the diamond represented the predicted target of each active ingredient.

**FIGURE 4:**
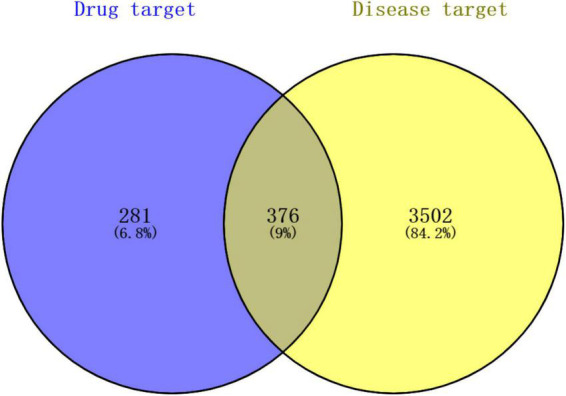
Venn diagram of the targets of the active ingredients of KXS and the depression-related targets. Blue bubble shows the potential targets of KXS, the yellow bubble represents the targets of depression. The overlapping part of the circles represents potential herbs target for depression therapy.

After deduplication, 3878 depression-related targets were obtained in the GeneCards, OMIM, and Disgenet databases. Six hundred and fifty-seven targets of the KXS were predicted to be related to depression. Finally, 376 targets were the same as drugs and diseases (Figure 4), which were the final predicted targets for treating depression with KXS.

**FIGURE 5:**
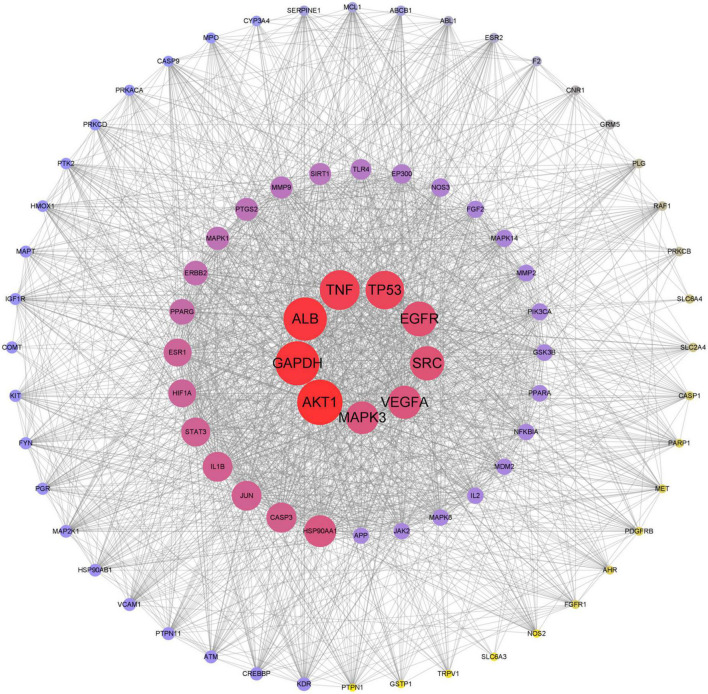
Protein-protein interaction (PPI). The larger and darker circle in the center indicated that these genes were more critical in the treatment of depression with KXS.

The intersection genes were imported into the protein interaction analysis platform String, the episomal node was hidden, and the PPI relationship diagram was obtained (Figure 5). The larger and darker circles in the center (AKT1, GAPDH, ALB, TNF, TP53, EGFR, SRC, VEGFA, and MAPK3) indicated that these genes were more critical in the treatment of depression with KXS.

### Gene ontology function and Kyoto encyclopedia of genes and genomes enrichment analysis

**FIGURE 6:**
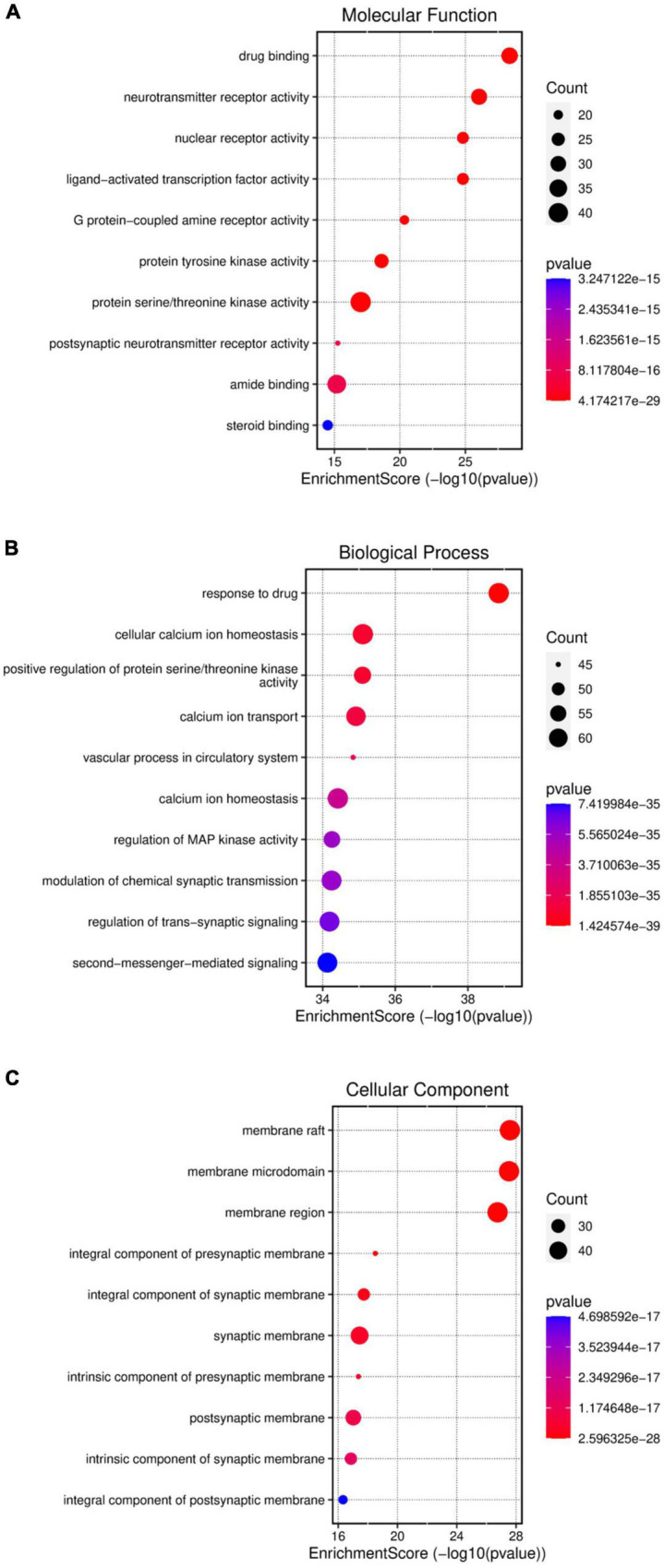
Gene ontology (GO) function. The GO function includes MF **(A)**, BP **(B)**, and CC **(C)**.

**FIGURE 7:**
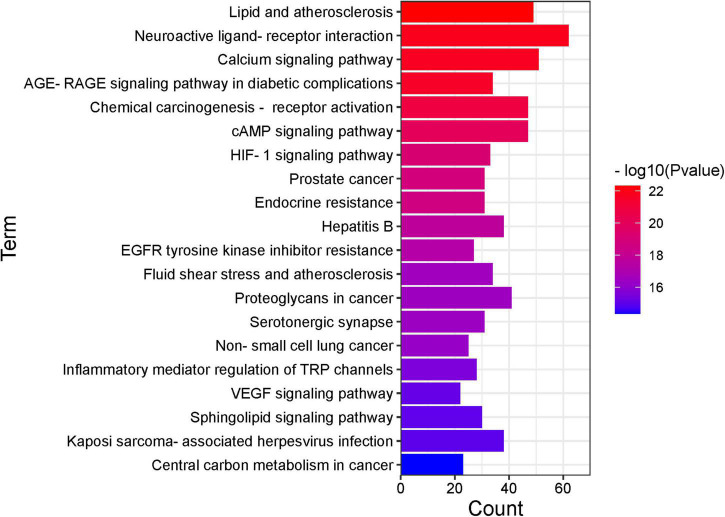
Kyoto encyclopedia of genes and genomes (KEGG) pathway (top 20). The x-axis represents the number of target genes in each pathway and the ordinate represents each entry.

Gene ontology function and KEGG enrichment analysis were performed on 376 intersecting targets, and the enrichment condition was *P* < 0.05. The GO function includes three parts: molecular function (MF), biological process (BP), and cellular component (CC). Target genes in MF were mainly enriched in drug binding (GO: 0008144), neurotransmitter receptor activity (GO: 00.30594), nuclear receptor activity (GO: 0004878), and ligand-activated transcription factor activity (GO: 0098531) (Figure 6A). The top five terms in the biological processes were: response to drug (GO: 0042493), cellular calcium ion homeostasis (GO: 0006874), positive regulation of protein serine/threonine kinase activity (GO: 0071902), calcium ion transport (GO: 0006816), and vascular process in the circulatory system (GO: 0003018) (Figure 6B). The most common cellular components of target genes were the membrane raft (GO: 0045121), the membrane microdomain (GO: 0098857), and the membrane region (GO: 0098589) (Figure 6C). KEGG enrichment analysis showed that KXS in the treatment of depression mainly exerted its effects through lipid and atherosclerosis, neuroactive ligand-receptor interaction, calcium signaling pathway, and chemical carcinogenesis-receptor activation (Figure 7).

## Discussion

Using the research method of network pharmacology to analyze traditional Chinese prescription, the single corresponding relationship of “one disease-one drug-one gene” has been broken, and the efficient utilization of multiple targets and effects between drugs and disease has been identified (Shen et al., 2020).

The efficacy of KXS in the treatment of depression was assessed in a meta-analysis using sucrose consumption and 5-HT level in a depression model as indicators. Seventy mice were included in eight studies for sucrose preference analysis, four studies evaluated 5-HT levels, and 38 animals were included. The results of the study showed a positive effect in the treatment of depression with KXS. Nevertheless, this study also had some shortcomings. One concern was that the number of animals included was relatively limited.

The PPI network shows that the core targets of KXS in treating depression were AKT1, GAPDH, ALB, TNF, TP53, EGFR, SRC, VEGFA, and MAPK3. AKT1 is one of the serine/threonine kinases, which participates in monoamine neurotransmission (Machado-Vieira et al., 2015). Additionally, there was a significant correlation between the severity of depression and the AKT1 gene polymorphisms. One theory was that AKT1 was closely related to bipolar disorder (BD), a mental genetic disease characterized by depression and mania. AKT1 was a first-line target for treatment of BD (Beaulieu, 2012). Although a recent cohort study contradicted the relationship between AKT1 and BD (Millischer et al., 2020), the correlation with the occurrence of depression was certain. AKT1 participates in the myelination of the peripheral nervous system and neurotrophic protein signaling pathway: the AKT/mTOR pathway may be the common core target of psychoactive drugs (Musashe et al., 2016; Liu et al., 2018). GAPDH, tumor necrosis factor (TNF), and TP53 were closely related to apoptosis. The GAPDH level in blood was positively correlated with Alzheimer’s disease (AD; Tsai et al., 2020). TNF induce low-grade inflammation, and inflammatory cytokines were the receptor regulators of 5-HT, which were related to the pathogenesis of anxiety and major depression (Schmidt et al., 2019; Karadag et al., 2021). TP53 was involved in astrocyte autophagy and neuronal apoptosis, which was the antidepressant mechanism of fluoxetine (Shu et al., 2019).

The lipid and atherosclerosis pathway in KEGG analysis was one of the key pathways in the treatment of depression with KXS. Omega-3 polyunsaturated fatty acids in lipids, including eicosapentaenoic acid (EPA) and docosahexaenoic acid (DHA), play an important role in antidepression and neuroprotection (Borsini et al., 2021). The lack of DHA may cause depression (Hsu et al., 2018). Furthermore, some studies showed that plasma lipoprotein was potentially related to the aggravation of depressive symptoms (Hui et al., 2021).

Patients with AD have an increased social burden due to cognitive and emotional disorders. As the second most common mental symptom of AD, depression increases the disability rate of AD patients. Prior studies have demonstrated the usefulness of KXS for AD (Yi et al., 2020), indeed, KXS reduced oxidative stress through acetylcholine, leading to improved cognitive function (Xu et al., 2019). This study explored the positive effects and mechanisms of KXS on emotions from the perspective of mental health. The research results helped promote the recovery of AD patients in many aspects. The majority of network pharmacological research about antidepression drugs focused on safety because of their diverse reactions (Cipriani et al., 2018; Cortese et al., 2018; Zhou et al., 2020), but KXS, as a Chinese medicine prescription with higher safety, is more focused on the exploration of the promotion mechanism.

This study was mainly based on big data analytics. Further pharmacological experimental evidence is needed to investigate the antidepressant effect of KXS. Moreover, the evaluation of the efficacy of KXS in this study was limited to animal models. Although an accurate animal model can reproduce the pathophysiological changes of human diseases, the uncertainty and repeatability of depression increase the difficulty to cure the disease, and more clinical studies should be actively carried out to the premise of ensuring safety.

## Conclusion

We explored the treatment of depression with the traditional Chinese prescription, KXS, by integrating meta-analysis and network pharmacology. Our study proved that KXS is capable of systematically improving depression symptoms through multi-target and multi-pathway systems. Elucidation of the components of the pathway and core targets with a high likelihood for translation into therapies for depression. Multi-pathways will not only greatly increase the multitarget effect, but overcome resistance and adverse effects. Therefore, the present findings may have important implications for clinical studies.

## Data availability statement

The original contributions presented in this study are included in this article/Supplementary material, further inquiries can be directed to the corresponding authors.

## Author contributions

ZL and GW designed the research. HZ and XJ did the proofreading. JX performed the image production and editing. MB and HJZ collected and analyzed the data and wrote the manuscript. All authors contributed to the article and approved the submitted version.

## Footnotes

1
http://tcmspw.com/tcmsp.php

2
https://pubchem.ncbi.nlm.nih.gov

3
http://www.swisstargetprediction.ch

4
http://www.uniprot.org/

5
https://www.genecards.org/

6
https://www.omim.org/

7
https://www.disgenet.org/search

8
https://bioinfogp.cnb.csic.es/tools/venny/

9
https://string-db.org/

10
https://david.ncifcrf.gov/
