# Supplementary material for: Systematic review of Kaixinsan in treating depression: Efficacy and pharmacological mechanisms
Source: Front Behav Neurosci. 2022 Dec 6;16:1061877. doi: 10.3389/fnbeh.2022.1061877 (PMC9763288; doi:10.3389/fnbeh.2022.1061877)
Supplement: Supplementary file 1 [file Data_Sheet_1.docx]

**Search strategy used in PubMed database**

＃1 kaixinsan [MeSH]

＃2 kai-xin-san [tiab]

＃3 traditional Chinese medicine [tiab]

＃4 animal trials [tiab]

＃5 trial [ti]

＃6 ＃1 OR ＃2 OR ＃3 OR ＃4 OR ＃5

＃7 depression [MeSH]

＃8 depressive disorders [MeSH]

＃9 mood disorders [tiab]

＃10 AD [tiab]

＃11 ＃7 OR ＃8 OR ＃9 OR ＃10

＃12 ＃6 and ＃11

**Search strategy used in CNKI database**

＃1 开心散 [MeSH]

＃2 抑郁症 [MeSH]

＃3 ＃1 and ＃2
